# Immunosuppressive effects of tick protein RHcyst-1 on murine bone marrow-derived dendritic cells

**DOI:** 10.1186/s13071-019-3411-1

**Published:** 2019-04-15

**Authors:** Nana Wei, Zhibing Lin, Zhengmao Xu, Haiyan Gong, Houshuang Zhang, Yongzhi Zhou, Jie Cao, Guoqing Li, Jinlin Zhou

**Affiliations:** 1 Key Laboratory of Animal Parasitology of Ministry of Agriculture, Shanghai Veterinary Research Institute, Chinese Academy of Agricultural Sciences, Shanghai, China; 2 Guangdong Provincial Zoonosis Prevention and Control Key Laboratory, College of Veterinary Medicine, South China Agricultural University, Guangzhou, China

**Keywords:** Tick, RHcyst-1, Cystatin, Dendritic cells, Immune response

## Abstract

**Background:**

Ticks, as blood-feeding arthropod vectors, have evolved their own unique mechanism to suppress host immune responses and evade immune defenses in order to complete blood-feeding. The immunoregulatory effect of tick bioactive molecules on hosts has been widely reported, and the cystatin family has been identified as one of the major immunomodulators. In previous studies, we obtained a novel tick salivary bioactive protein named RHcyst-1, which belongs to the type 1 cystatin family. Here, we demonstrated the effects of RHcyst-1 on the host immune response mainly on dendritic cell (DC) function. Understanding the function of tick-derived bioactive molecule may help to clarify the mechanisms of how ticks escape the host immune response and help to control ticks and tick-borne disease transmission.

**Methods:**

Bone marrow-derived DCs (BMDCs) were generated and induced by GM-CSF and IL-4 with or without RHcyst-1 addition. Flow cytometry was used to analyze the differentiation and maturation of BMDCs and T cell cytokine production. Quantitative real-time PCR (qRT-PCR) and western blot were used to measure changes in expression within STAT and p38 MAPK signaling pathways.

**Results:**

Flow cytometry analysis revealed that RHcyst-1 inhibited the differentiation of BMDCs, but had no effect on the maturation of BMDCs. T cells co-cultured with DCs treated with RHcyst-1 produced significantly less TNF-α, IFN-γ and IL-2 than the control group. Further analysis showed that the mRNA level and phosphorylation of p38, ERK and STAT were significantly changed after RHcyst-1 added to bone marrow monocytes during the differentiation stage.

**Conclusions:**

Our results suggest that RHcyst-1 is one of the major immunosuppressive proteins of BMDC function from blood-feeding ticks.

**Electronic supplementary material:**

The online version of this article (10.1186/s13071-019-3411-1) contains supplementary material, which is available to authorized users.

## Background

Ticks are obligate hematophagous ectoparasites. During blood-feeding, ticks transmit multiple pathogenic agents which can cause different zoonoses, such as fever with thrombocytopenia syndrome, rickettsiosis, Lyme disease, tick-borne encephalitis, ehrlichiosis, and human granulocytic anaplasmosis [1]. In order to finish the blood meal and facilitate pathogen transmission, ticks produce many bioactive molecules, which contribute to ticks evading surveillance from the host immune system. These bioactive molecules have anti-hemostatic, anti-inflammatory, anti-complement and immunomodulatory properties. These bioactive molecules contain multiple proteases and protease inhibitors, among which cysteine protease inhibitors such as cystatins represent one of the most prominent protein families [2, 3].

Cystatins are reversible and tight-binding inhibitors of cysteine proteases and they are distributed in almost all organisms [4]. Cystatins are divided into three subgroups, including type 1 (stefins), type 2 (cystatins) and type 3 (kininogens) [5]. Cystatins can regulate many biological processes, including antigen processing/presentation, phagocytosis, inflammation, T cell proliferation and tumor progression [6, 7]. Cystatin F is expressed primarily in immune cells and is an important regulator of cell cytotoxicity [8]. Cystatin SA induces IFN-γ expression in CD4+ T cells [9]. In human tissues and cells, cystatin C is the most abundant cystatin and may modulate the function of different immune cells [10]. In ticks, to date, only type 1 and type 2 cystatins have been reported, and more studies have been focused on type 2 cystatin in ticks because of their secretory nature [3]. Tick salivary cystatin OmC2 compromises human DC function by targeting cathepsins S and C [11]. DsCystatin, a novel immunosuppressive protein, inhibits TLR2 and TLR4-directed NFκB activation and also alleviates joint inflammation in mouse models of *Borrelia burgdorferi*- or complete Freund’s adjuvant-induced arthritis [12].

Cystatin *RHcyst*-*1* (GenBank: KM588364), belonging to the type 1 cystatin family, has been detected in embryos, larvae, nymphs and adults, but is most highly expressed at the embryo stage. The RNAi of cystatin *RHcyst*-*1* decreases the rate of embryo hatching. It has been reported that RHcyst-1 has a broad spectrum of inhibitory activities against mammalian cysteine proteases [13]. DCs are the most powerful antigen-presenting cells (APCs) and are key regulators in the immune system. In DCs, mammalian cysteine proteases, especially cathepsins, play important roles in antigen processing and presentation [14]. In this study, we show that RHcyst-1, identified from *Rhipicephalus haemaphysaloides*, significantly inhibits BMDC differentiation from precursors and impairs the production of cytokines. Hence, RHcyst-1 is an effective immunosuppressive molecule, which helps ticks to evade the host immune response.

## Methods

### Experimental animals

C57BL/6 mice (4–6 weeks of age) were purchased from Slac Laboratory Animal, Inc. (Shanghai, China) and maintained in a SPF facility under sterile conditions at the Animal Center of the Shanghai Veterinary Research Institute (IACUC approve number shvri-mo-2018010019). All animal experiments were performed with permission from local animal-ethics committees.

### RNA extraction and qRT-PCR experiments

Total RNA was extracted from different tick organs or cells using TRIzol^®^ (Life Technologies, CA, USA). RNA was reverse transcribed into cDNA using Prime Script™ RT Reagent with genomic DNA removal (Takara, Dalian, China) by following the manufacturer’s recommendations. Next, qRT-PCR was performed using SYBR^®^ Premix Dimmer Eraser kit (Takara) for 40 amplification cycles at 95 °C for 30 s, 95 °C for 5 s and 60 °C for 34 s. The relative expression level of *RHcyst*-*1* in different tick organs was evaluated using the 2^−ΔCt^ method. ELF1α was used as the internal control. ERK, p38, STAT3, STAT4 and STAT5 transcription were evaluated using the 2^−ΔΔCt^ method [15]. Mouse β-actin, a housekeeping gene, was used as the internal control. All qRT-PCR amplifications were performed in triplicate with three independent experiments. All qRT-PCR primers used in this study are listed in Table 1.

**Table 1:** Primer sequences used in qRT-PCR analysis. F and R indicate forward and reverse primers, respectively

Gene name	Primer sequence (5′-3′)	Length (bp)
*RHcyst-1*	F: CACAGTCAGGGAGATTTGCG	96
	R: TGCGTGCGATACTTCAGAGG	
*ELF1α*	F: CGTCTACAAGATTGGTGGCATT	108
	R: CTCAGTGGTCAGGTTGGCAG	
*STAT3*	F: CACCTTGGATTGAGAGTCAAGAC	112
	R: AGGAATCGGCTATATTGCTGGT	
*STAT4*	F: GCAGCCAACATGCCTATCCA	97
	R: TGGCAGACACTTTGTGTTCCA	
*STAT5*	F: CGCCAGATGCAAGTGTTGTAT	113
	R: TCCTGGGGATTATCCAAGTCAAT	
*p38*	F: CTGACCGACGACCACGTTC	118
	R: CTTCGTTCACAGCTAGGTTGC	
*ERK*	F: CAGGTGTTCGACGTAGGGC	139
	R: TCTGGTGCTCAAAAGGACTGA	
*Mus-actin*	F: AGAGGGAAATCGTGCGTGAC	195
	R: CCATACCCAAGAAGGAAGGCT	

### Immunofluorescence assay (IFA)

After dissection in PBS, salivary glands were fixed with 4% paraformaldehyde, dehydrated in a graded series of ethanol and embedded in paraffin. Five micrometer sections were cut and mounted on glass slides. The paraffin was removed from the sections with xylene and the sections were hydrated by successive 5-min washes with a graded series of 100, 85 and 75% ethanol and distilled water. Fifteen minutes of antigen retrieval by microwave with ETDA buffer solution (pH 8.0) was performed, followed by washing with PBS three times. The slides were blocked with 3% BSA for 30 min at room temperature. Then, the slides were incubated overnight at 4 °C with primary antibodies and after three washes in PBS, followed for 1 h with goat-anti-mouse IgG conjugated with FITC (Thermo Fisher, Waltham, MA, USA). The slides were washed with PBS and stained with 10 mg/ml DAPI for 5 min at room temperature (Sigma-Aldrich, St. Louis, MO, USA). The sections were imaged using a confocal laser-scanning microscope (Zeiss LSM 880, Oberkochen, Germany). The parameters for all the experimental and control groups were set at the same values.

### Generation of BMDCs

BMDCs were generated according to the previously described procedure by Inaba et al. [16], with some modifications. Briefly, bone marrow-derived monocytes from tibias and femurs of C57BL/6 mice were cultured in RPMI-1640 complete medium supplemented with 10% FBS (Gibco, Grand Island, NY, USA), 100 U/ml penicillin (Gibco) and 100 μg/ml streptomycin (Gibco). On day 0, 5 × 10^6^ cells were seeded into 100-mm dishes (Corning, Corning, NY, USA) in 10 ml RPMI-1640 complete medium plus 40 ng/ml GM-CSF (Pepro Tech, Rocky Hill, NJ, USA) and 20 ng/ml IL-4 (Pepro Tech). On day 3, another 10 ml of RPMI-1640 complete medium plus 20 ng/ml GM-CSF was added to the dish. On days 6 and 8, half of the supernatants were removed and replaced with 10 ml fresh complete medium with sufficient GM-CSF and IL-4. On day 9, non-adherent cells were collected and used as immature BMDCs.

To analyze the effects of two cystatins on BMDC differentiation, highly purified RHcyst-1 (1.8 μM) was added into the above isolated cells beginning on day 0 of culture and the cells were harvested on day 3 and day 6 and analyzed by flow cytometry (Beckman CytoFLEX, Miami, FL, USA). To analyze the effects of RHcyst-1 on BMDC maturation, the immature BMDCs were activated by the addition of 1 μg/ml LPS (Sigma-Aldrich) with or without RHcyst-1 treatment. After 18 h of incubation, the cells were harvested and the expression of CD40, CD80, CD86 and MHC class II molecules was analyzed by flow cytometer.

### T cell isolation and co-culture with DCs

C57BL/6 mice T cells from spleens were isolated according to the Mouse spleen T Cell Isolation Kit’s instructions (TBD sciences, Tianjin, China). Differentiated BMDCs with GM-CSF and IL-4, as described above were collected on day 9 and incubated with 1 mg/ml OVA (GL Biochem, Shanghai, China) with or without RHcyst-1 (1.8 μM) treatment for 24 h. DCs were co-cultured with T cells from spleen in the presence of 1 mg/ml OVA with or without RHcyst-1 treatment for another 72 h. To determine cytokine production in DC and T cell co-cultures by flow cytometry, PMA, Ionomycin (R&D Systems, Minneapolis, MN, USA) and protein transport inhibitors (BD, Franklin Lakes, NJ, USA) were used. Then, the cells were collected for cytokine analysis by flow cytometry.

### Flow cytometry analysis

For cell-surface staining, cells with different treatments were collected and washed with PBS, and then incubated with FcR-blocking reagent for 20 min at 4 °C. The cells were then washed and stained by using the following mAbs (BD): anti-CD11b-FITC, anti-CD11c-APC, anti-CD40-PB450, anti-CD80-PE, anti-CD86-PE, anti-MHC-II-PB450, anti-IFN-γ-APC, anti-TNF-α-PB450, anti-IL-2-PE and isotype-matched mAbs for control staining. Finally, the cells were resuspended in 100 μl of staining buffer for flow cytometry analysis (Beckman CytoFLEX). Data were analyzed with FlowJo software (Tree Star, Ashland, OR, USA).

### Western blot

Cells were harvested and lysed with IP buffer (Beyotime, Suzhou, China) for 20 min on ice and centrifuged for 10 min (12,000 rpm, 4 °C). Protein concentrations were quantified using a BCA protein assay kit (Thermo Fisher). Then, 20–50 μg total proteins from each sample were electrophoresed in a 12% SDS-AGE gel and transferred onto polyvinylidene fluoride (PVDF) membranes (Millipore, Billerica, MA, USA). The membranes were blocked with 5% nonfat milk for 1 h at 37 °C and probed with anti-phosphorylated or unphosphorylated p38 MAPK, p44/42 MAPK (Erk1/2), STAT3 and STAT5 antibody (CST, Danvers, MA, USA) overnight at 4 °C. The bone marrow-derived monocyte induced with GM-CSF/IL-4 stimulation without RHcyst-1 treatment was used as negative control. The membranes were washed with TBST and then probed with secondary antibody goat anti-mouse or goat anti-rabbit IgG (Thermo Fisher) for 1 h at 37 °C. Band signals were visualized with Pierce™ ECL Western Blotting Substrate (Thermo Fisher) under a Multiple-color Fluorescence Imaging System (Bio-Rad, Hercules, CA, USA).

### Statistical analysis

All statistical analyses were performed using GraphPad Prism 6 software (GraphPad Software Inc., La Jolla, CA, USA). Numerical data are presented as the mean ± SD, and most of the data were analyzed by Student’s t-test. In all statistical analyses, * indicates *P* < 0.05, ** indicates *P* < 0.01, *** indicates *P* < 0.001 and NS indicates no significance.

## Results

### Expression analysis of RHcyst-1

**Fig. 1:**
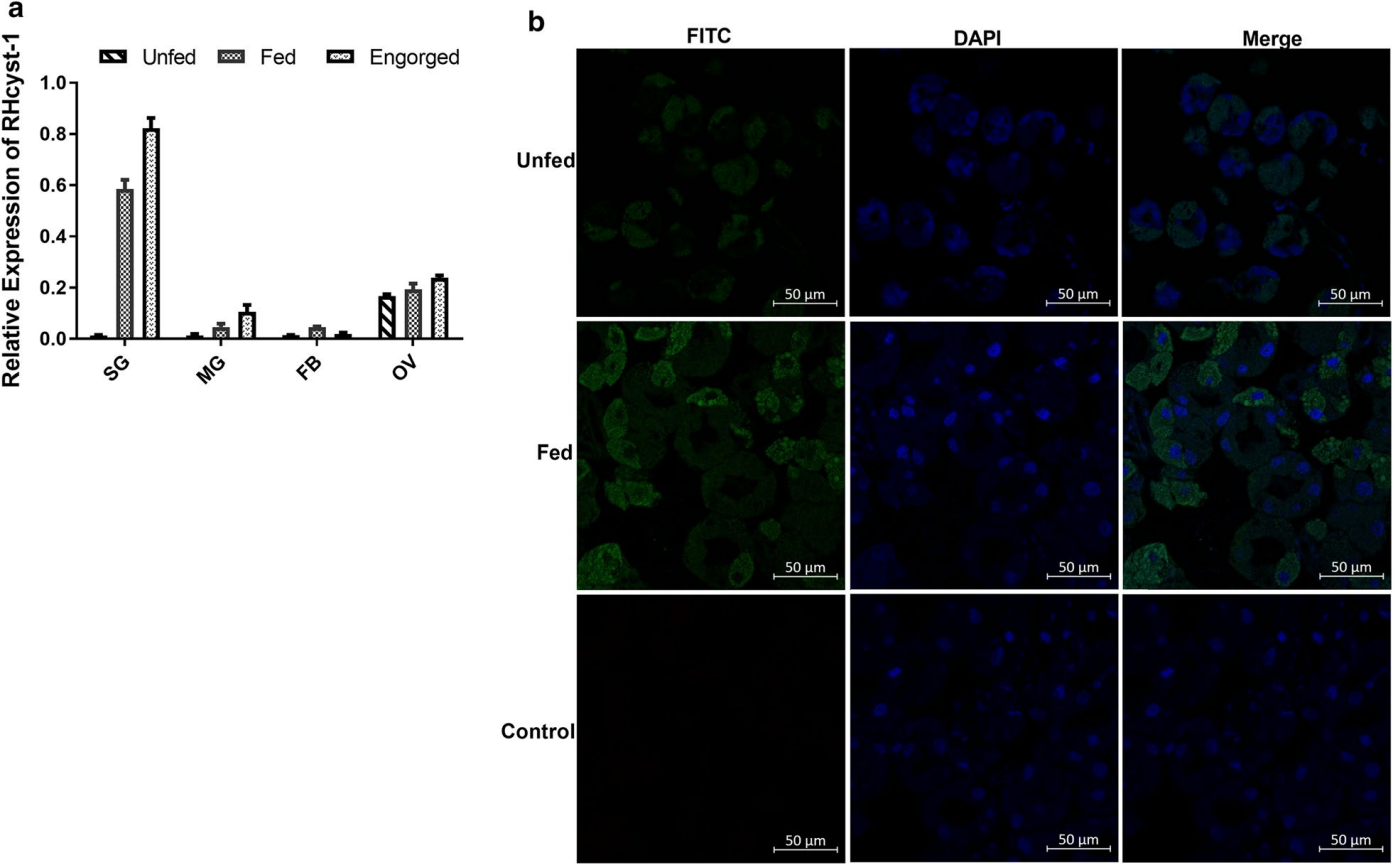
The expression and distribution of RHcyst-1. **a** Gene expression patterns of *RHcyst*-*1*, isolated from *Rhipicephalus haemaphysaloides*, in different organs from unfed, fed and engorged adult ticks using qRT-PCR. **b** Distribution of native RHcyst-1 in SG from unfed and fed adult ticks. Bars represent the mean ± SD. Data are representative of three independently repeated experiments. *Abbreviations*: SG, salivary glands; MD, midgut; FB, fat body; OV, ovary

To determine the expression profile of *RHcyst*-*1*, total RNA from adult ticks’ salivary gland (SG), midgut (MD), fat body (FB), ovary (OV) were subjected to qRT-PCR testing. The primers used in this study are shown in Table 1. As shown in Fig. 1a, *RHcyst*-*1* was highly expressed in the SG, and the expression in the SG was upregulated from unfed to engorged ticks. Furthermore, we investigated the distribution of RHcyst-1 in the SG of unfed and fed ticks using IFA. We found that RHcyst-1 was highly expressed in the SG during blood-feeding (Fig. 1b). These results suggest that RHcyst-1 is a salivary gland protein and may play an important role during tick blood-feeding.

### RHcyst-1 impairs differentiation of BMDC

**Fig. 2:**
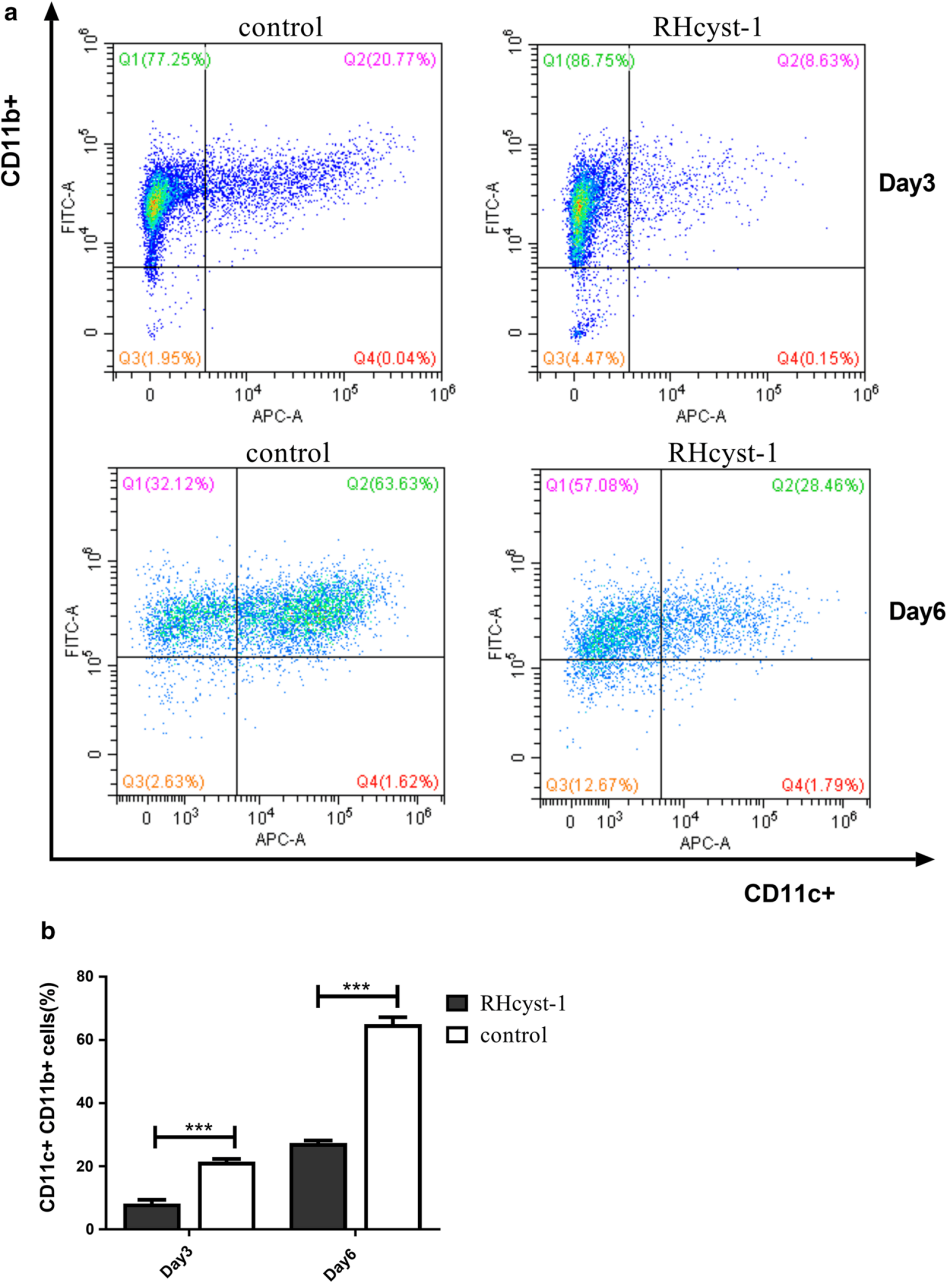
RHcyst-1 modulates the differentiation of bone marrow-derived monocytes into DCs. Bone marrow monocytes were cultured in GM-CSF and IL-4 condition (control) or in the presence of RHcyst-1. CD11c+ CD11b+ cells were analyzed by flow cytometry. **a** Differentiated cells (CD11c+ CD11b+) are shown in dot plots. **b** The representative of three independent experiments is shown. *** indicates *P* < 0.001; bars represent the mean ± SD

Tick salivary gland proteins involved in host immune responses have been widely reported. To explore the effect of RHcyst-1 on host immune responses, we focused on DC function. DCs are pivotal APCs, which bridge innate and adaptive immune systems and are the initiator and modulator of the immune response [17]. To search for the effect of RHcyst-1 on the differentiation of DCs, bone marrow-derived monocytes were induced with GM-CSF/IL-4 in the presence or absence of RHcyst-1, respectively. In order to verify whether the effect was directed against an early or a late phase of BMDC differentiation, the cells were collected on the third and sixth day and the ratio of BMDCs was evaluated by flow cytometry. As shown in Fig. 2, RHcyst-1 significantly inhibited the differentiation of bone marrow monocytes into CD11c+ CD11b+ BMDCs, whether it was on day 3 (RHcyst-1: 8.16 ± 0.70% *vs* control: 21.44 ± 0.50%; t-test: *t*_(4)_ = 15.4, *P* = 0.0001) or day 6 (RHcyst-1: 27.37 ± 0.43% *vs* control: 64.97 ± 1.28%; *t*_(4)_ = 27.75, *P* = 0.00001).

In addition, the safety of RHcyst-1 on the normal cell lines 293T, Vero and CHO has been evaluated in our previous studies. RHcyst-1 treatment did not change the activity of normal cells and did not induce apoptosis in these cell lines [18]. These results show that the suppression of BMDC differentiation by RHcyst-1 is not due to apoptosis or cytotoxicity.

### RHcyst-1 shows no effect on BMDC maturation

**Fig. 3:**
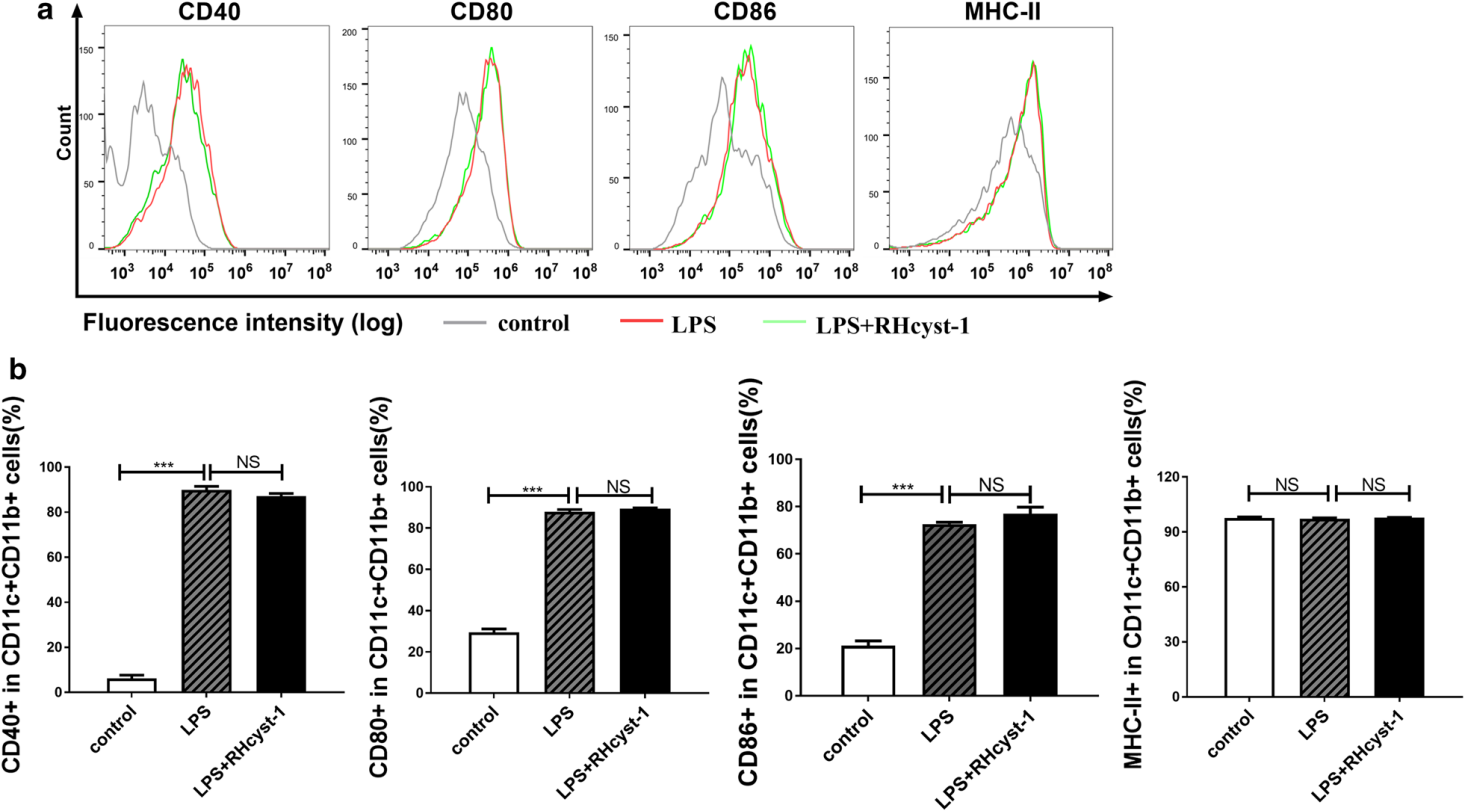
RHcyst-1 shows no effect on LPS induced BMDC maturation. BMDCs obtained from bone marrow monocytes culture for 9 days were stimulated with LPS (1 μg/ml) alone or in combination with RHcyst-1 (1.8 μM) for 18 h. Cells were collected and stained. The surface expression of CD40, CD80, CD86 and MHC-II were analyzed by flow cytometry. **a** FACS histograms from one representative experiment. **b** The percentage of co-stimulatory and MHC-II molecules expression on CD11c+ CD11b+ cells. One-way ANOVA was applied and a *P*-value < 0.05 was considered statistically significant. *** indicates *P* < 0.001 and NS indicates *P* > 0.05; bars represent the mean of three independent experiments ± SD

In the following experiments, we analyzed the effect of RHcyst-1 on LPS- stimulated maturation of BMDCs. Bone marrow monocytes were cultured in the presence of GM-CSF and IL-4 for 9 days, and the differentiated immature DCs were harvested. The harvested BMDCs were stimulated by LPS for 18 h with or without RHcyst-1. Then, the BMDCs were stained and analyzed for the expression of CD40, CD80, CD86 and MHC-II molecules by flow cytometry. The immature DCs stimulated with LPS showed significantly increased expression of CD40 (one-way ANOVA: *F*_(2,6)_ = 3477, *P* = 0.00001) CD80 (*F*_(2,6)_ = 2477, *P* = 0.00001) and CD86 (*F*_(2,6)_ = 697.5, *P* = 0.00001) compared with that of the control group. However, there was no change on LPS-induced upregulation of CD40 (one-way ANOVA: *F*_(2,6)_ = 3477, *P* = 0.0953), CD80 (*F*_(2,6)_ = 2477, *P* = 0.3282), CD86 (*F*_(2,6)_ = 697.5, *P* = 0.0638) and MHC-II (*F*_(2,6)_ = 1.805, *P* = 0.1841) when BMDCs were exposed to RHcyst-1 (Fig. 3). These results indicate that RHcyst-1 has no effect on DC maturation.

### RHcyst-1 inhibits cytokine levels in BMDC

**Fig. 4:**
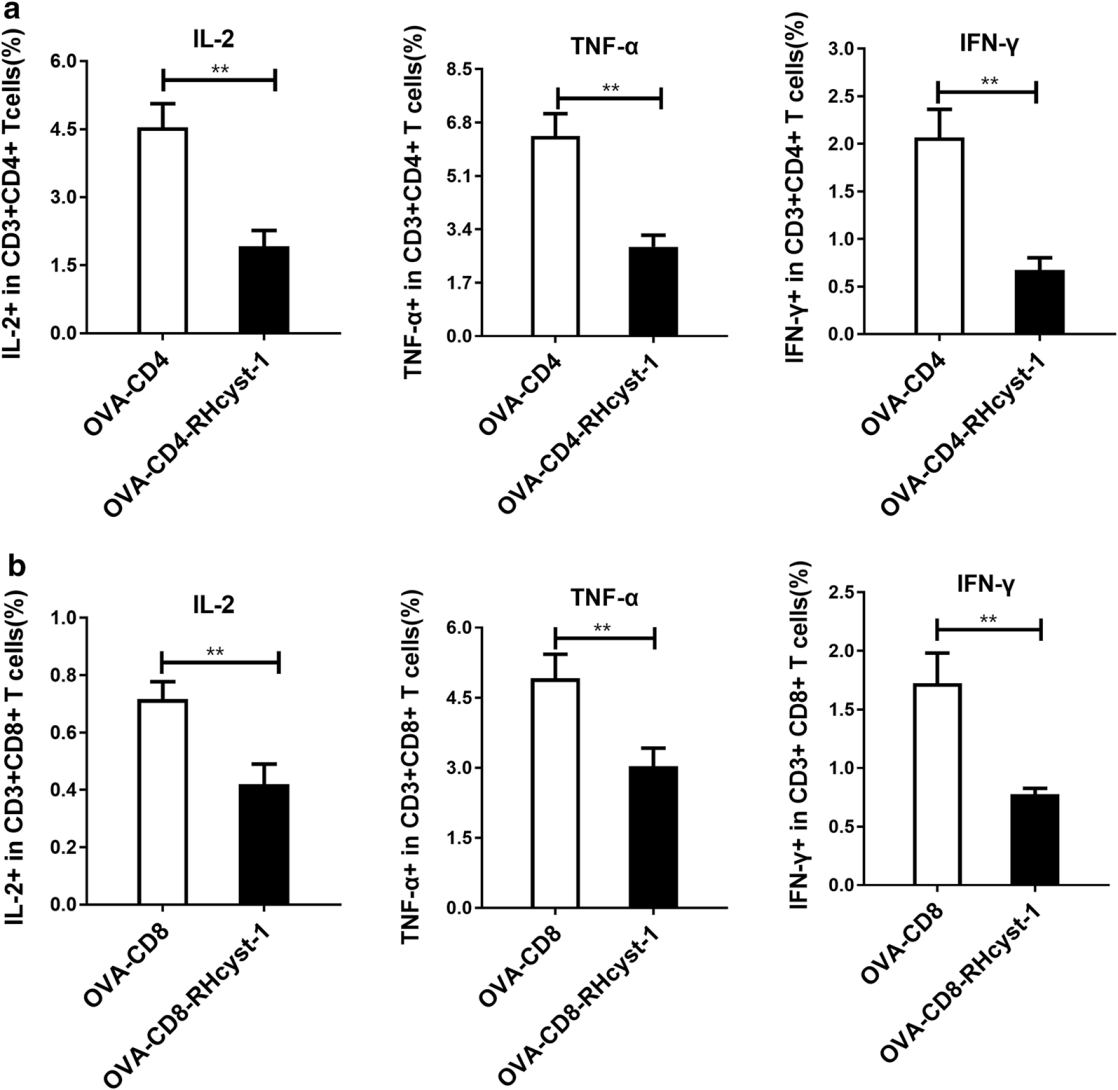
RHcyst-1 inhibits T cell activation and cytokine production. BMDCs were stimulated with OVA-CD4 (**a**) and OVA-CD8 (**b**) peptide with or without RHcyst-1 treatment, respectively. The above BMDCs were co-cultured with T cells from spleen for another 72 h. The levels of IL-2, TNF-α and IFN-γ were measured by flow cytometry. Student’s t-test was applied and a *P*-value < 0.05 was considered statistically significant. ** indicates *P* < 0.01; bars represent the mean of three independent experiments ± SD

The levels of cytokines are critical in the regulation of DC function because of their ability to activate T cell responses. Thus, we analyzed cytokine levels in RHcyst-1-treated BMDCs in response to OVA stimulation. As shown in Fig. 4a, b, a significant reduction was detectable following RHcyst-1 treatment for IL-2, TNF-α and IFN-γ, after OVA-CD4 (t-test: *t*_(4)_ = 7.918, *P* = 0.002; *t*_(4)_ = 7.665, *P* = 0.0016; *t*_(4)_ = 7.539, *P* = 0.0017) and OVA-CD8 (t-test: *t*_(4)_ = 5.563, *P* = 0.0051; *t*_(4)_ = 5.056, *P* = 0.0072; *t*_(4)_ = 6.257, *P* = 0.0033) peptide stimulated, respectively. In conclusion, RHcyst-1 inhibits the level of cytokines which are important for T cell activation.

### Cell signaling pathways induced by RHcyst-1 during BMDC differentiation

**Fig. 5:**
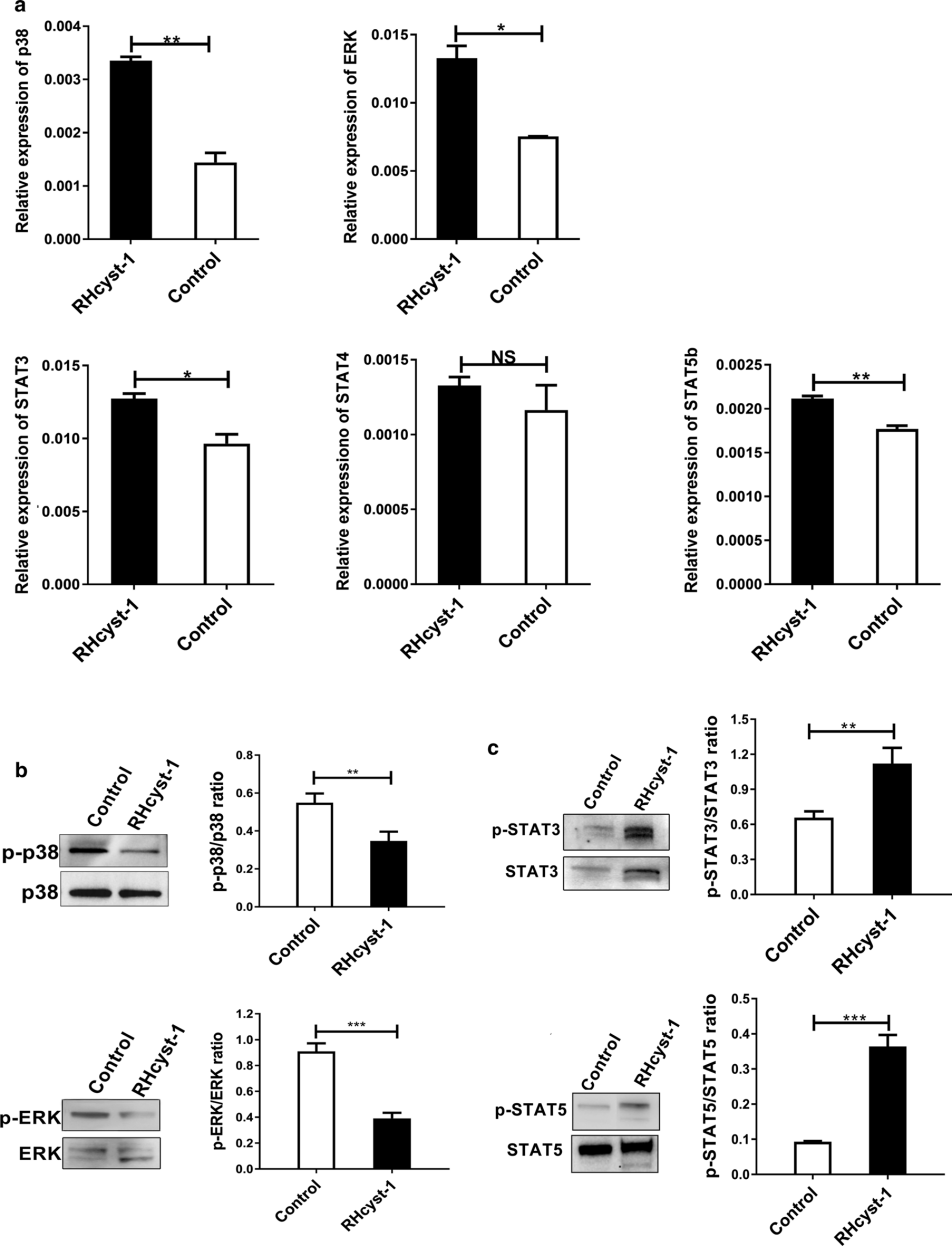
RHcyst-1 affects p38 MAPK and STAT signaling pathways during the differentiation BMDCs. **a** The mRNA expression of p38 MAPK and STAT signaling pathways during BMDC differentiation after RHcyst-1 treatment was quantified by qRT-PCR. The effect of RHcyst-1 on phosphorylation of p38 MAPK pathway (**b**) and STAT pathway (**c**) during BMDCs differentiation was determined by western blot. Student’s t-test was applied and a *P*-value < 0.05 was considered statistically significant. *P < 0.05, **P < 0.01, ***P < 0.001 and NS indicates *P* > 0.05; bars represent the mean ± SD

The p38 MAPK signaling pathway is crucial for the differentiation of BMDC [14]. Combined inhibition of STAT3 and p38 MAPK could prevent tumor-mediated suppression of monocyte-derived DC differentiation [19]. To examine signaling pathways of RHcyst-1 involved in bone marrow monocytes differentiated into DCs, cells from day 6 cultures were prepared for western blot analyses. As shown in Fig. 5a, the mRNA levels of p38 (t-test: *t*_(2)_ = 13.92, *P* = 0.0051), ERK (*t*_(2)_ = 8.964, *P* = 0.0122), STAT3 (*t*_(2)_ = 6.01, *P* = 0.0266) and STAT5 (*t*_(2)_ = 10.44, *P* = 0.0091) were all upregulated compared to those of the control group. The primer sets used in this study are shown in Table 1. We also tested the phosphorylation levels of p38 MAPK and STAT signaling pathways. As shown in Fig. 5b, c, decreased pp38 (t-test: *t*_(4)_ = 5.061, *P* = 0.0072) and pERK (*t*_(4)_ = 11.96, *P* = 0.0003) levels and increased pSTAT3 (*t*_(4)_ = 5.53, *P* = 0.0052) and pSTAT5 (*t*_(4)_ = 14.25, *P* = 0.0001) levels were observed in cells treated with RHcyst-1 compared with those in the control group. These results show that RHcyst-1 inhibits the p38 MAPK pathway and activates the STAT signaling pathway during the differentiation of BMDC.

## Discussion

Immunosuppression that occurs during the process of tick blood-feeding has been reported extensively [20–23]. Saliva and salivary gland extracts are involved in the modulation of host immunity [24]. Tick salivary gland extracts are composed of multiple proteins belonging to different protein families, such as the cystatin family, serpin family and Kunitz inhibitor family. In this study, we found that RHcyst-1 is highly expressed in salivary glands and upregulated during tick blood-feeding (Fig. 1a, b). Our results demonstrate the immune inhibitory properties mainly on DC function of a salivary gland cystatin, RHcyst-1, isolated from the tick *Rhipicephalus haemaphysaloides.*

DCs are powerful antigen-presentation cells and are essential in initiating adaptive immune responses in hosts [17]. DCs can also directly contact and affect pathogen infection and be targeted by ticks to suppress the initiation of adaptive immune responses in hosts [25, 26]. In this study, we demonstrate that RHcyst-1 significantly inhibits the differentiation of BMDC and suppresses DC-induced T-lymphocyte activation. There have been some previous reports about tick cystatins on DC function [2, 27, 28]. However, how tick cystatin regulates the host immune signaling pathway and impairs the host immune cell function remains largely unclear.

Many tick cystatins belonging to type 1 and 2 families have been reported. Studies of the function of tick cystatins in the host have primarily focused on the type 2 family [2]. In our previous study, we reported that RHcyst-1 belongs to the type1 cystatin family and is involved in tick physiology [13]. In this study, although RHcyst-1 was not directly detectable in serum from rabbits on which ticks had repeatedly fed (Additional file 1: Figure S1), previous studies have shown that RHcyst-1 has a broad spectrum of mammalian cathepsin-inhibition activities and mediates specific immune responses in tumor-bearing mice [13, 18]. Recently, exosomes have been most extensively studied and tick-derived exosomes were reported to mediate transmission of tick-borne disease [29–32]. Whether RHcyst-1, as the component of exosomes, enters into the host to regulate the host immune response remains to be further investigated.

Protein phosphorylation is a modification of proteins and is the major molecular mechanism of protein regulation. The STAT and p38 MAPK pathways are tightly associated with DC differentiation [14, 19]. In the present study, we found that RHcyst-1, a cysteine protease inhibitor, inhibited the phosphorylation of p38 and ERK, but increased the phosphorylation of STAT3 and STAT5 during BMDC differentiation. It has been reported that cystatin from the murine nematode parasite, *Heligmosomoides polygyrus*, alters the T cell activating functionality of DCs [33]. In this study, in addition to changes in STAT and p38 MAPK signaling pathways, we observed that RHcyst-1 treatment suppressed T cell activation by BMDCs. The observations made in our study may represent one of the important mechanisms by which blood-feeding ticks induce immunosuppression in their hosts. Regulating DC differentiation and function may be a potential strategy for ticks to promote blood-feeding and pathogen transmission. More effort is being done to study the critical roles of biologically important tick bioactive molecules in tick-pathogen-hosts [34–36]. RHcyst-1 may be a promising target for tick control in the future.

## Conclusions

We show here that the tick type 1 cystatin, RHcyst-1, affects different functions of BMDCs. In particular, RHcyst-1 significantly inhibits BMDC differentiation, in part through p38 MAPK inhibition and STAT activation. Our results contribute to clarifying tick-host interactions and provide a better understanding of the mechanisms used by tick bioactive molecules to escape host immune system detection and allow ticks to successfully feed.

## Additional file

**Additional file 1: Figure S1.** Western blot analysis of RHcyst-1. Total 20 μg of purified RHcyst-1 was electrophoresed in SDS–PAGE gel and then transferred onto PVDF membrane for Western blot analysis with serum from rabbits on which ticks had repeatedly fed (a) and normal rabbit serum as control (b).

## Abbreviations

DC: dendritic cell
BMDC: bone marrow-derived DC
APCs: antigen-presenting cells
ERK: extracellular-regulated protein kinases
MAPK: mitogen-activated protein kinase
STAT: signal transducers and activators of transcription
cystatin: cysteine protease inhibitor
ELF1α: elongation factor-1 gene
PBS: phosphate-buffered saline
OVA: ovalbumin
TNF-α: tumor necrosis factor-alpha
IFN-γ: interferon-gamma
IL-2: interleukin-2
IL-4: interleukin-4
GM-CSF: granulocyte-macrophage colony stimulating factor
