# Supplementary material for: Immunosuppressive effects of tick protein RHcyst-1 on murine bone marrow-derived dendritic cells
Source: Parasit Vectors. 2019 Apr 15;12:169. doi: 10.1186/s13071-019-3411-1 (PMC6466765; doi:10.1186/s13071-019-3411-1)
Supplement: Supplementary file 1 — Additional file 1: Figure S1. Western blot analysis of RHcyst-1. Total 20 μg of purified RHcyst-1 was electrophoresed in SDS–PAGE gel and then transferred onto PVDF membrane for Western blot analysis with serum from rabbits on which ticks had repeatedly fed (a) and normal rabbit serum as control (b). [file 13071_2019_3411_MOESM1_ESM.docx]

**Additional file 1: Figure S1** Western blot analysis of RHcyst-1. Total 20 μg of purified RHcyst-1 was electrophoresed in SDS–PAGE gel and then transferred onto PVDF membrane for Western blot analysis with serum from rabbits on which ticks had repeatedly fed (a) and normal rabbit serum as control (b).
